# Author Correction: Competition between crystal and fibril formation in molecular mutations of amyloidogenic peptides

**DOI:** 10.1038/s41467-017-02141-8

**Published:** 2017-12-20

**Authors:** Nicholas P. Reynolds, Jozef Adamcik, Joshua T. Berryman, Stephan Handschin, Ali Asghar Hakami Zanjani, Wen Li, Kun Liu, Afang Zhang, Raffaele Mezzenga

**Affiliations:** 1 Swinburne University of Technology, ARC Training Centre for Biodevices, Faculty of Science, Engineering and Technology, John Street, Melbourne, VIC 3122 Australia; 2 ETH Zurich, Department of Health Science & Technology, Schmelzbergstrasse 9, LFO, E23, 8092 Zürich, Switzerland; 3 University of Luxembourg, Department of Physics and Materials Science, 162a Avenue de la Faïencerie, Luxembourg City, L-1511 Luxembourg; 4 Shanghai University, Department of Polymer Materials, Nanchen Street 333, Shanghai, 200444 China; 5 ETH Zurich, Department of Materials, Wolfgang-Pauli-Strasse 10, 8093 Zurich, Switzerland

Correction to: *Nature Communications* 10.1038/s41467-017-01424-4, published online: 20 Jul 2017

The original version of this article contained an error in Fig. 5c. The label for the back series of columns was incorrectly given as ‘1.5 mM pH 2’, rather than the correct ‘1.5 mM pH 7’. This has now been corrected in both the PDF and HTML versions of the article.

