# Supplementary material for: Competition between crystal and fibril formation in molecular mutations of amyloidogenic peptides
Source: Nat Commun. 2017 Nov 7;8:1338. doi: 10.1038/s41467-017-01424-4 (PMC5673901; doi:10.1038/s41467-017-01424-4)
Supplement: Supplementary file 1 — Description of Additional Supplementary Files [file 41467_2017_1424_MOESM1_ESM.pdf]

Description of Additional Supplementary Files

File Name: Supplementary Movie 1

Description: Time series of cross-sectional shape distributions for aggregating 3 peptides. The peptides for which aggregation on the a axis is initially suppressed eventually 4 form more large, long and shape-monodisperse aggregates (length is annotated as Lz), 5 destined to grow into crystalline structures. Time and concentration (color map) are 6 represented using a logarithmic scale.

File Name: Supplementary Movie 2

Description: This movie shows the softest normal mode of the ILQINS pH7 8 simulation trajectory. The structure flexes with saddle-like curvature. The normal mode was 9 extracted using principal components analysis of the simulation trajectory. Only C alpha 10 atoms are shown.

File Name: Supplementary Movie 3

Description: This movie shows the ensemble of unit cells extracted from the 12 ILQINS pH7 simulation for the purpose of scattering analysis. The ensemble is sorted with 13 respect to the unit cell angular parameter [illegible]) the most variable of the unit cell parameters, 14 both between and within systems simulated). The global deformation of changing saddle- 15 like curvature (Supplementary Movie 2) is associated locally with variation in [illegible] ,which is 16 realised mainly by shear at the hydrophobic interface.
