# Supplementary material for: Competition between crystal and fibril formation in molecular mutations of amyloidogenic peptides
Source: Nat Commun. 2017 Nov 7;8:1338. doi: 10.1038/s41467-017-01424-4 (PMC5673901; doi:10.1038/s41467-017-01424-4)
Supplement: Supplementary file 3 — Supplementary Information [file 41467_2017_1424_MOESM3_ESM.pdf]

Supplementary Methods:

Peptide Synthesis

The hexapeptides were all synthesized by standard solid phase peptide synthesis from Wangresin with O-(benzotriazole-1-yl)-1,1,3,3-tetramethylcarbamide tetrafluoroborate (TBTU) as the coupling reagent and N,N' diisopropylethylenamine (DiPEA) as the base. 1-Hydroxybenzotriazole (HOBt) was used to avoid intramolecular cyclization to form diketopiperazine. A typical synthetic procedure was: After swelling the resin in DMF overnight, Fmoc-protected amino acid (4 equiv), TBTU (4 equiv), DiPEA (4 equiv) and HOBt (4 equiv) in DMF were added and shaken. After coupling for 1 h, the resin was washed with DMF (4 Å~ 1 min) and DCM (4 Å~ 1 min). Removal of the Fmoc group was performed with piperidine (15 min). The peptide was cleaved from the resin with HF in the presence of 10% anisole for 1 h at 0 ^{o}C. The crude peptide was precipitated with anhydrous *tert*-butylmethyl ether, dissolved in AcOH and lyophilized, which was further purified by RP-HPLC with gradients of water and acetonitrile. For ILQINS the molecular weight of 686.8 Da measured was found to be in good agreement with expected mass of 686.4 Da. (Mw IFQINS = 720.82 Da, TFQINS = 708.76 Da).

Self-assembly of hexapeptides

The lyophilized hexapeptides were mixed with MilliQ water at the desired concentrations (either 1.5 or 5 mM) to form solutions of the chosen hexapeptide which due to the low buffering capability of ultrapure water were found to possess a pH that varied between 6 and 7 (for simplicity termed pH 7 throughout). Alternatively, the peptide was added to a

solution of 996 µl MilliQ water with 4 µl of 1 M HCl added (resulting in a solution of hexapeptide at pH2). Self-assembly took place at room temperature.

Self-assembly of R3 peptide

The R3 fragment (VQIVYKPVDLSKVTSKCGSLGNIHHK), a 26-mer fragment from Tau protein, was purchased from CS Bio Co. (Menlo Park, CA, USA). The peptide was dissolved in a buffered solution (10 mM Tris, 50 mM NaF, 0.5 mM dithiothreitol, pH 7.4) at 200 µM. The buffered R3 solutions were then incubated for one week at 37 ^{o}C.

Transmission Electron Microscopy

Transmission electron microscopy (TEM) analysis was performed using a Tecnai 12 Transmission Electron Microscope (FEI, Eindhoven, The Netherlands) at an operating voltage of 120 kV. Images were recorded using a Megaview III CCD camera and AnalySIS camera control software (Olympus). Carbon-coated 300-mesh copper grids were glow- discharged in nitrogen to render the carbon film hydrophilic. A 4 µL aliquot of the sample was pipetted onto each grid. After 30 s adsorption time, the excess was drawn off using Whatman 541 filter paper, followed by staining with 2% aqueous potassium phosphate at pH 7.2, for 10 s. Grids were air-dried until needed.

cryo-Transmission Electron Microscopy

200-mesh copper grids coated with a perforated carbon film (Lacey carbon film: ProSciTech, Qld, Australia) were used for all experiments. Grids were cleaned by glow discharge in Nitrogen for 5 seconds immediately before use. Solutions of assemblies were pipetted (4 µL)

onto the copper grids and allowed to adsorb to the grids for 30 s. After adsorption excess solution was removed from the grids by blotting with Whatmann 541 filter paper, for approximately 6−10 s. The adsorbed fibrils were cryo-frozen by plunging the grid into liquid ethane. The frozen grids were stored in liquid nitrogen until required. TEM was performed using a Gatan 626 cryoholder (Gatan, Pleasanton, CA, USA) and a Tecnai 12 transmission electron microscope (FEI, Eindhoven, The Netherlands) at an operating voltage of 120 kV. An electron dose of 8−10 electrons per $Å^2$ was used for all imaging. Images were recorded using a FEI Eagle 4k×4k CCD camera at using magnifications in the range 40 000× to 110 000×.

Electron diffraction

Diffraction patterns were imaged using a FEI Tecnai F20 transmission electron microscope (TEM). One drop of undiluted dispersion was placed on a carbon-coated grid (Quantifoil, D) previously glow-discharged for 20 s (Emitech K100X, GB) and allowed to adsorb for 60 s; after this interval excess fluid was removed by touching the edge of the grid with filter paper. Dried grids were transferred on a Gatan cryo-holder into the microscope and cooled down below -150 °C prior to the imaging process. Micrographs were recorded under low dose conditions ($< 10e^- / Å^2$) using a 4k x 4k Gatan CCD camera operating the microscope at 200 kV acceleration voltage in bright field mode and for SAD diffraction in low dose diffraction mode.

Atomic Force Microscopy

Aliquots of 20 µL, taken at different times during peptide incubation, were incubated for 2 min on freshly cleaved mica or on highly oriented pyrolytic graphite (HOPG), rinsed with

Milli-Q water, and dried with air. Images were collected using a Nanoscope VIII Multimode Scanning Force Microscope (Bruker) operated in tapping mode in air. Images were flattened using the NanoscopeAnalysis 8.15 software, and no further image processing was done. The statistical analysis of fibrils was performed by FiberApp software.[1]

Wide-Angle X-ray Scattering

WAXS was used to produce spectra of pre-assembled hexapeptides (assembled for approximately 24 hours, both at pH 7 and pH 2). Additional WAXS spectra were recorded after 60 seconds for each hexapeptide. Experiments were performed at room temperature at the SAXS/WAXS beamline at the Australian synchrotron. Samples were loaded into a 96 well plate held on a robotically controlled x-y stage and transferred to the beamline via a quartz capillary connected to a syringe pump. The experiments used a beam of wavelength of λ = 1.03320 Å (12.0 KeV) with dimensions 300 µm x 200 µm and a typical flux of 1.2 $x10^{13}$ photons per second. 2D diffraction images were recorded on a Pilatus 1M detector. Experiments were performed at q ranges between 0.03-1.5 $Å^{-1}$. Spectra were recorded at under flow (0.15 ml min^{-1}) in order to prevent X-ray damage from the beam. Multiples of 15 spectra were recorded for each time point (exposure time = 1 s) and the averaged spectra are shown after background subtraction against MQ water or MQ water at pH 2 in the same capillary.

Computational Scattering Calculations

The simulations carried out for the purpose of thermodynamic calculations were of size 1024 peptides x 25 ns; however in order to calculate scattering amplitudes a larger variety

of aggregate conformations than reachable through simple molecular dynamics was found to be needed. Observing the simulation trajectories, it was noted that the unit cell angle γ, fluctuated strongly, while a, b and c were relatively stable, so in order to generate a large ensemble of conformationally different structures for the purpose of scattering calculations, 3000 unit cells were sampled from different timepoints and lattice positions of the ILQINS pH7 trajectory. The unit cells were sorted by their associated values of γ, ranging from 74° to 91°, and 50 representative unit cells were then chosen so as to span an even sampling of this range. Each of the 50 selected unit cells was then constructed into a nanocrystal of 1024 peptides (8 x 8 x 8 cells), and relaxed in explicit water for 3 ns (with solute heavy atoms restrained) then analysed using crysol.[2] For the crysol analysis, scattering from water inside the nanocrystal was calculated explicitly. Water outside the nanocrystal was modelled as an infinite uniform medium of electron density 0.334 e/Å^{3}. The corresponding procedure was carried out for IFQINS and TFQINS, however this was done using the ILQINS unit cell parameter range as the IFQINS and TFQINS simulations did not show sufficient fluctuation over the simulation timescale. The match of IFQINS or TFQINS peptide structures to ILQINS unit cells was achieved by finding the closest peptide conformation from the appropriate simulation to the corresponding ILQINS unit cell internal conformation.

Detailed Simulation Methods

We define and explore a model for self-assembly of nanometre sized cuboidal particles (or parallelepipeds, without loss of generality) in aqueous solution. Each face of the cuboid may attach only to the opposite face of another such particle (so that in an aggregate, all particles have the same orientation), and each Cartesian axis of the cuboid (each unit-cell

boundary) is associated with a different interaction strength. The diffusing particles are taken to represent assembly-competent amyloidogenic peptides and the three pairs of faces taken to represent assembly in the terminus-terminus (a), sidechain-sidechain (b) or hydrogen-bonding (c) directions.

As the assemblies grow their speed of diffusion decreases, subject to a non-trivial dependence on aspect ratio. Counteracting the slowing transport, larger objects have a larger cross-section for collision and also the assembly-competent planes give a stronger adhesive energy with larger size. The resulting kinetic scheme is complex and best examined via numerical sampling, however the rates extracted can also serve as the basis of an approximate analytical description of the assembly process.

SYSTEM SUMMARY

We define monomeric units having axes $a, b, c = 2.17, 0.99, 0.49\ nm$. We also define per-axis free energies of assembly ΔG_a°, ΔG_b°, ΔG_c°. The values ΔG° are parameters of the model, however we note that for amyloidogenic peptides the free energy gain for assembly in c is considerably larger than for the other directions.

FORWARD ASSEMBLY RATE *VIA* DIFFUSION OF ANISOTROPIC BODIES

Given that assembly in c is considerably stronger than in a or b, leading to approximately rod-like aggregates, we employ the formula for diffusion of rod-like bodies given by Ortega & de la Torre.[3] In general the diffusion of anisotropic bodies is a difficult calculation due to the coupling of rotational and translational motion. We do not reproduce the formulae of Ortega & de la Torre here however, we can plot the calculated translational diffusion

constants for different sizes of aggregates (Supplementary Figure 1). We see that the shape has a real, but not an extremely large, impact on the speed of diffusion of the resulting object, and therefore on its probability to meet another object and assemble further before undergoing spontaneous fission.

To estimate the rate [measured in s^{-1}] for a collision of the a faces of some pair of bodies i, j drawn from populations of size N_i, N_j identical such objects we can write:

$$r_{aa} = k_{aa} \frac{N_i N_j}{V} \qquad (1)$$

$$k_{aa} = 2\, e^{-3}(D_i + D_j)\sqrt{2bL_b cL_c} \qquad (2)$$

where e^{-3} is a 'barrier' term representing an entropic cost of $3k_BT$ to join two rigid bodies (thereby removing six degrees of freedom from the system). We note that the free energy barrier for monomer addition to a growing amyloid fibril has been measured calorimetrically for many systems[4] and often found to be in the region of $3k_BT$. This is most likely the result of many terms cancelling rather than a genuine resemblance of the various different proteins and peptides examined to ideal rigid bodies. $(D_i + D_j)\sqrt{2bL_b cL_c}$ is the diffusive collision term, and the factor $\frac{2N_iN_j}{V}$ comes from the symmetry of assembly. Note that in the case that $i = j$ (homodimerisation), the symmetry factor is reduced to $N_i(N_i - 1)/V$.

For purposes of numerical simulation, assembly of non-matching planes is permitted with a rate penalty of $e^{-\Delta}$ where Δ represents the solvation free energy cost (see **Backwards Rate: Activated Fission**) needed to cleave the colliding aggregates so as to generate a pair of matching faces (and thus a joined object, as for a normal collision of matching planes, plus

one or two fragmentation products).

BACKWARDS RATE: ACTIVATED FISSION

To calculate a rate for the splitting processes, an energetic scale is necessary. We measure the energetic cost to create an interface in the a, b, c axes using atomistic simulation of a large (1024 peptide × 10 ns) microcrystal. If we denote the free energy of a $m \times n \times o$ block as G_{mno} then we can find the free energy per face of our cuboidal unit cell buried to create each interface as the difference between the master block energy and the summed energy of the blocks generated by fracturing on that plane, divided by the total number of unit-cell face pairs exposed by the fracture: $\Delta G^{\circ} = (G^{\circ}{}_{mno} - mG^{\circ}{}_{1no})/(m-1)no$.

$$(2\times6\times6)\Delta G_a^{\circ} = G_{366}^{\circ} - 3G_{166}^{\circ} \qquad (3)$$

$$(3\times5\times6)\Delta G_b^{\circ} = G_{366}^{\circ} - 6G_{316}^{\circ} \qquad (4)$$

$$(3\times6\times5)\Delta G_c^{\circ} = G_{366}^{\circ} - 6G_{361}^{\circ} \qquad (5)$$

Here the dimensions $3 \times 6 \times 6$ of the 'master' block are chosen so as to give a roughly square structure, given that $a \approx 2b$. Average energies were taken over 1000 master blocks sampled from the final nanosecond of each atomistic trajectory, and the blocks analysed using the molecular mechanics functionality of AMBER15.[5] The G and ΔG values found contain atomistic interaction enthalpies, and also estimated solvation enthalpies and entropies via the Generalised Born approximation.[6,7] The ΔG does not contain

configurational entropy changes, however we can note that these are likely to be very similar among peptide systems of similar sequence and assembled (parallel β-sheet) structure.

For the splitting process, a dynamic timescale τ_0 is necessary, and we pick the time for a single free peptide to diffuse its own length (calculated via the Ortega formula[3]) to serve this role. This gives an Arrhenius rate expression [measured in s^{-1}] to split, for example, on an a plane:

$$r_a = N k_a \qquad (6)$$

$$k_a = \frac{1}{\tau_0}(L_a - 1)\, e^{\frac{L_b L_c \Delta G^{\circ}_a}{k_B T}} \qquad (7)$$

Where N is the number present of the given species. From Supplementary Equation 7 we can see that growth in a increases the rate to split perpendicular to the a axis, as L_a is increased. Conversely, growth in b or c stabilises the a planes by increasing the area that must be cleaved. Permutation of the indices yields corresponding equations for b and c.

SIMULATION DETAILS:

Atomistic Simulation Details

Simulations were run using The University of Luxembourg high performance computing facility.[8] The AMBER simulations were run using the AMBER15 pmemd-cuda binary[5] and the AMBERFF14-SB all atom forcefield.[9] When the C-terminus was neutralised to mimic conditions of low pH, the same partial charges for the protonated C-terminal serine were

used as previously.[10] Temperature and pressure were held constant in the region of 300K/1atm using the weak-coupling algorithm.[11] The TIP3P water model was used.[12] Structures were based on the ILQINS pH2 structure previously validated against scattering,[10] with point-mutations carried out using pymol[13] without disruption to the unit-cell geometry.

Solvation Free Energy Calculation

The simulated microcrystal structures were subsampled in space and time to create 1000 $3 \times 6 \times 6$-peptide parallelepiped blocks using cpptraj.[14] The Hawkins, Cramer & Truhlar generalised Born model[15] was used to estimate the solvation energy of each block and block fragment. To reduce the impact of mismatches between explicit and implicit solvent energy landscapes, a short minimisation was carried out to find the local minimum for each block or sub-block analysed.

Kinetic Simulation Details

The aggregation process was simulated for systems of 1 million peptides using the event driven variant of the Doob-Gillespie algorithm.[16] Averages were collected over 20 calculations for each of the 6 peptide systems and two concentrations studied.

Supplementary Figures:

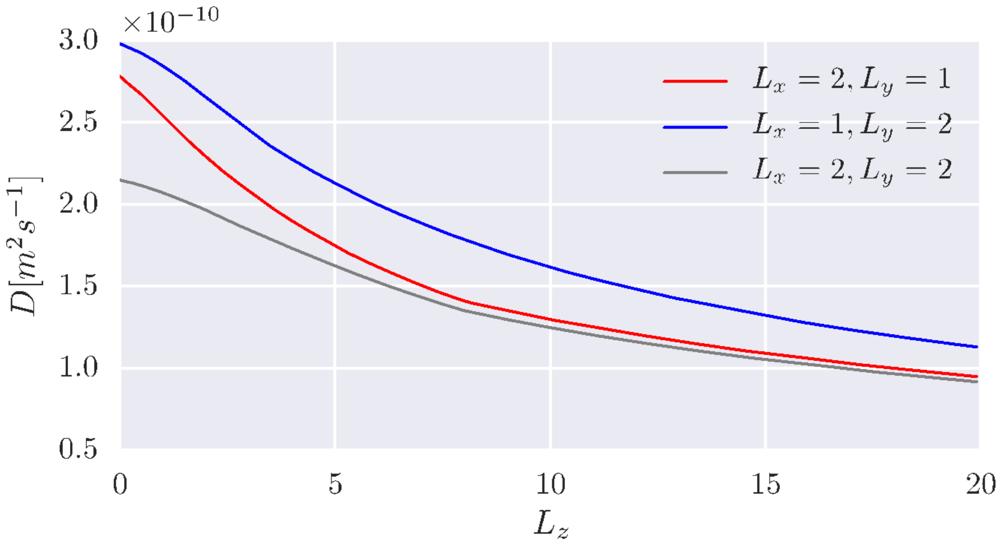

Supplementary Figure 1. Diffusion for anisotropic bodies. Assembly along the shorter axis ($b < a$) produces more rapidly-diffusing bodies as the resulting object has a smaller hydrodynamic radius, however the effect is of much less than an order of magnitude.

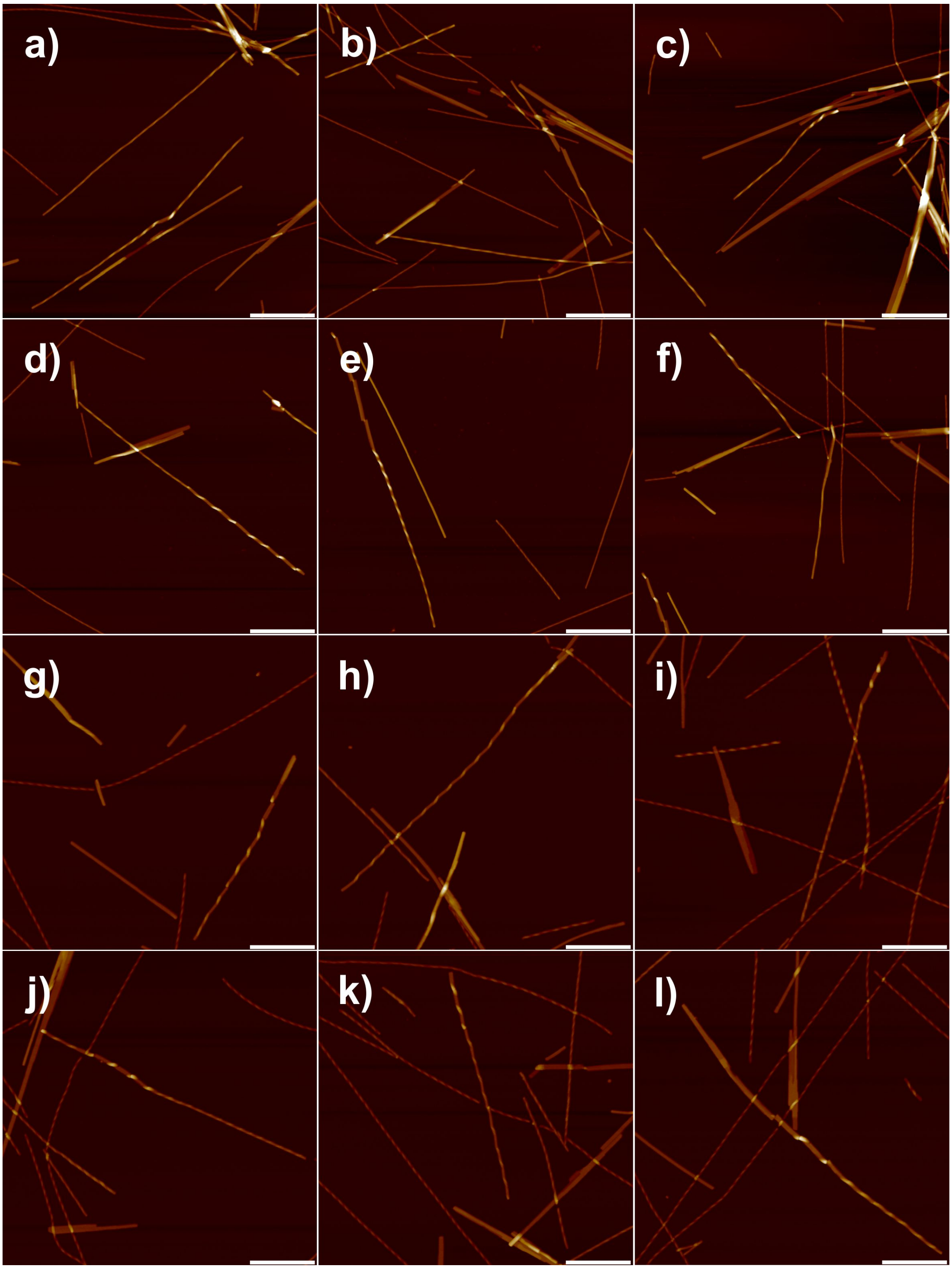

Supplementary Figure 2: Additional AFM images of IFQINS at pH 7 after 24 h incubation time deposited on mica. (a-c) Scale bars = 600 nm, (d-f) Scale bars = 600 nm, (g-i) Scale bars = 400 nm, (j-l) Scale bars = 400 nm, all Z-scales = 40 nm.

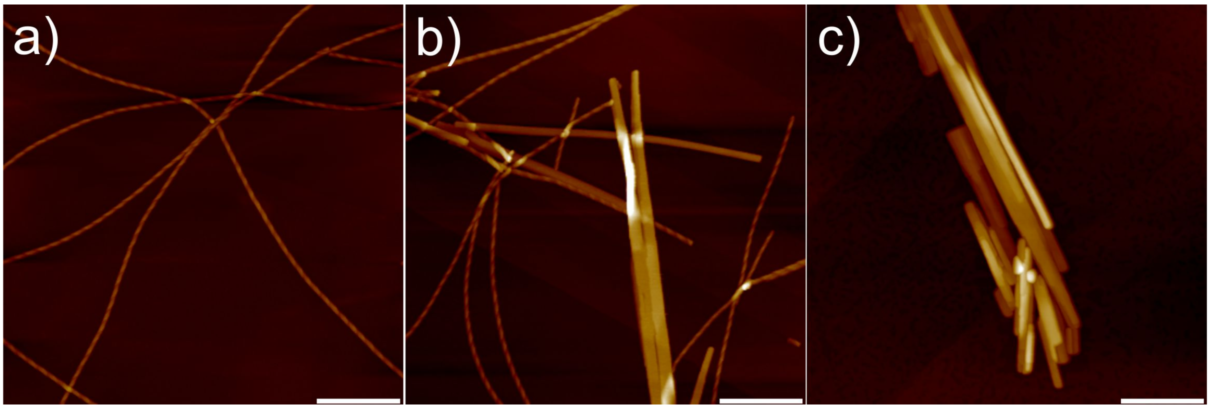

Supplementary Figure 3: AFM images of a) ILQINS fibrils, b) IFQINS fibrils and crystals, c) TFQINS crystals at pH 7 after 24 h incubation time deposited on a hydrophobic surface (HOPG). All scale bars = 400 nm, all Z-scales = 40 nm.

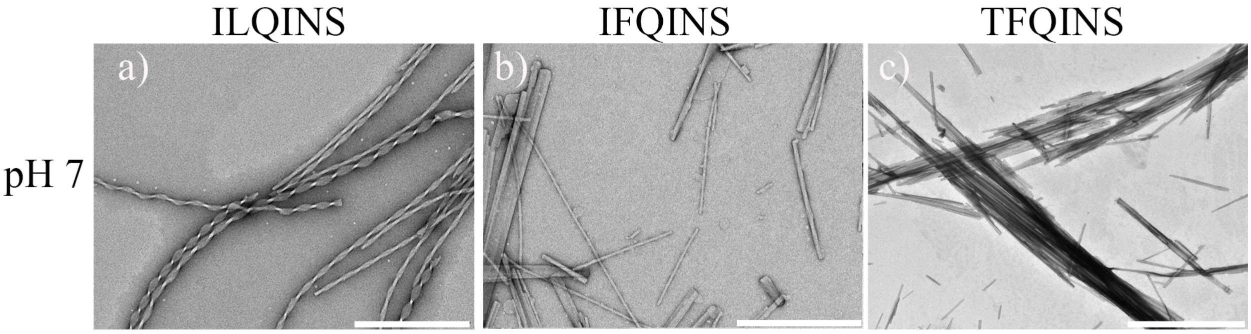

Supplementary Figure 4: Additional TEM images of a) ILQINS fibrils (scale bar = 500 nm), b) IFQINS fibrils and crystals (scale bar = 500 nm), c) TFQINS crystals at pH 7 (scale bar = 5 µm) after 24 h incubation time.

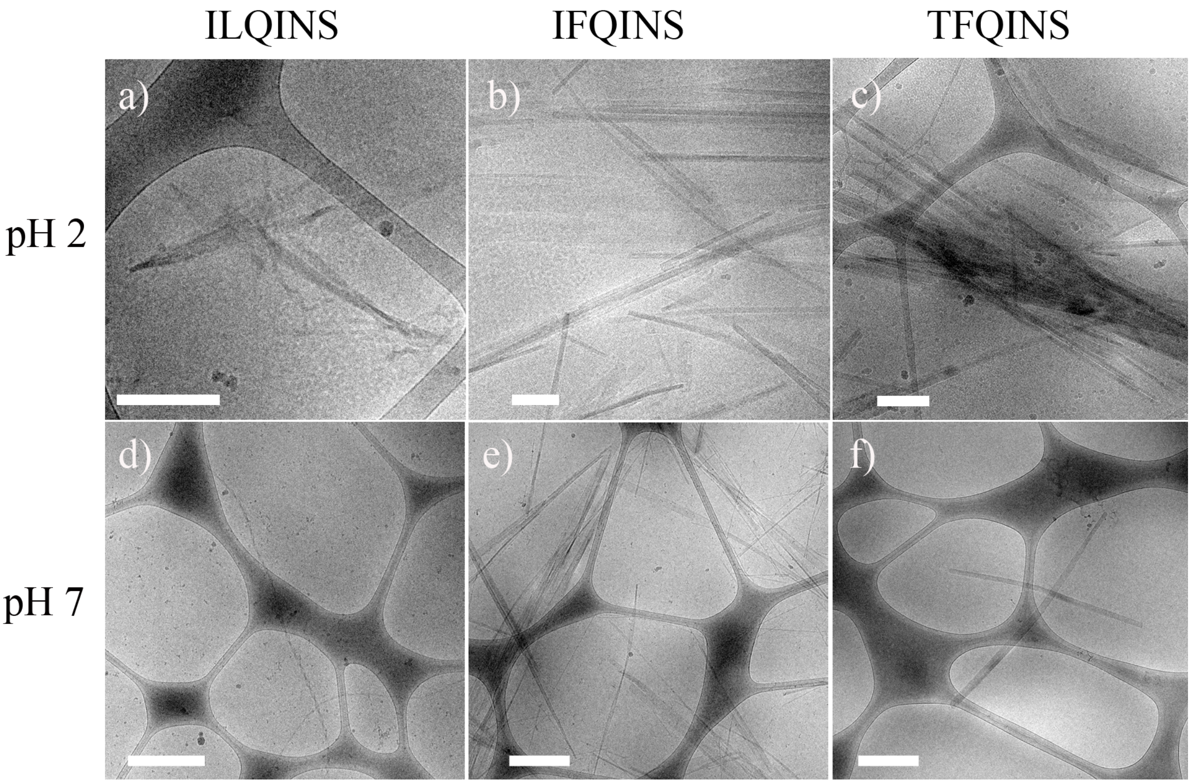

Supplementary Figure 5: cryo-TEM images of ILQINS, IFQINS and TFQINS after 24 h assembly. (a,b) scale bar = 200 nm, (c-e) scale bar = 500 nm, (f) scale bar = 1 µm.

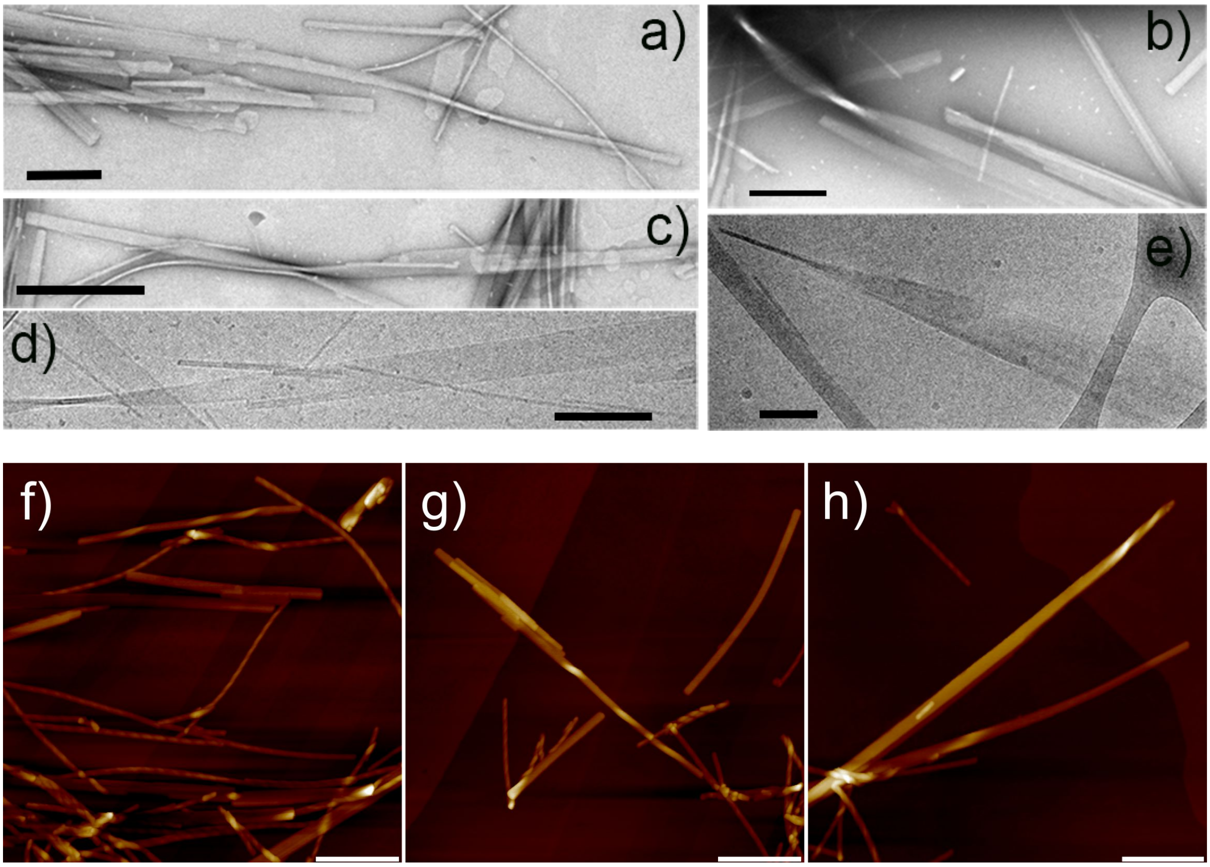

Supplementary Figure 6: Evidence of Fibril-Crystal Conversion for IFQINS at pH 7 on a carbon coated grid (negative stain, TEM) (a-c) scale bars = 250 nm, Substrate Free Conditions (cryo-TEM) (d, e) scale bars = 250 nm, and HOPG (AFM) (f-h) scale bars = 400 nm, z-scale = 40 nm.

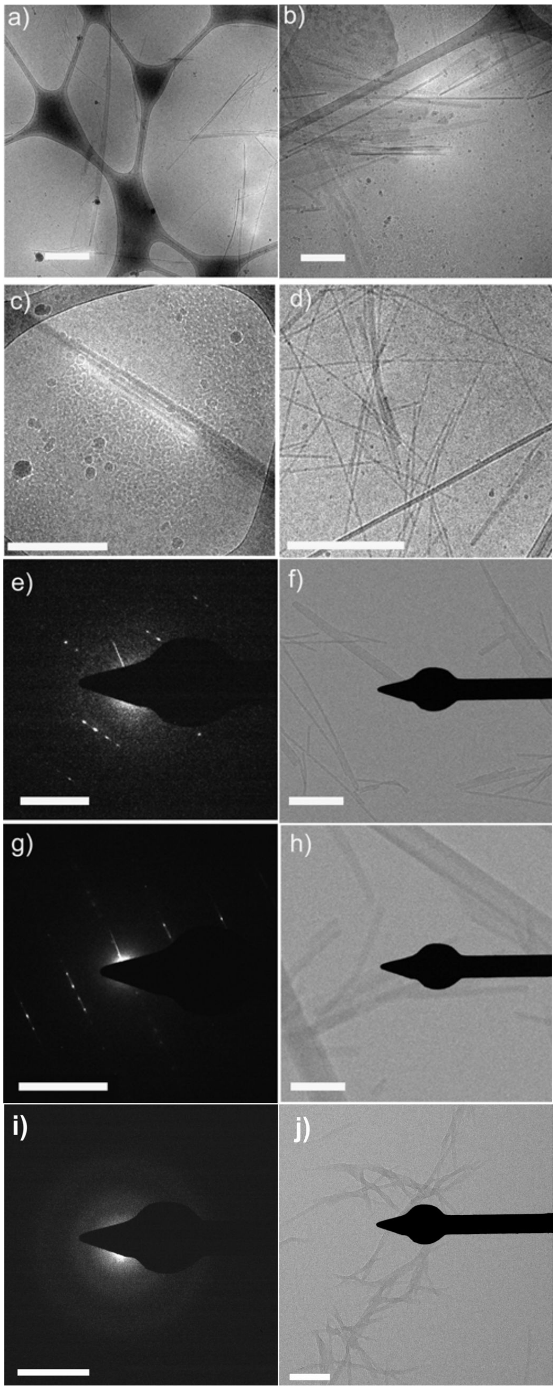

Supplementary Figure 7: a-d) Bright field cryoTEM of IFQINS, pH7 showing diffraction contrast arising from amyloid crystals (see a-d) and twisted ribbons in the process of crystal conversion (see a) but not well folded (helical) ribbons (see d). (a) scale bar = 500 nm, (b) scale bar = 200 nm, (c) scale bar = 250 nm, (d) scale bar = 500 nm. e) Selected area diffraction (SAD) of IFQINS, pH7 (scale bar = 20 nm^{-1}) with f) corresponding TEM image (scale bar = 200 nm). g) SAD of TFQINS, pH7 (scale bar = 20 nm^{-1}) with h) corresponding TEM image (scale bar = 200 nm). i) SAD of ILQINS, pH7 (scale bar = 100 nm^{-1}) with j) corresponding TEM images (scale bar = 200 nm).

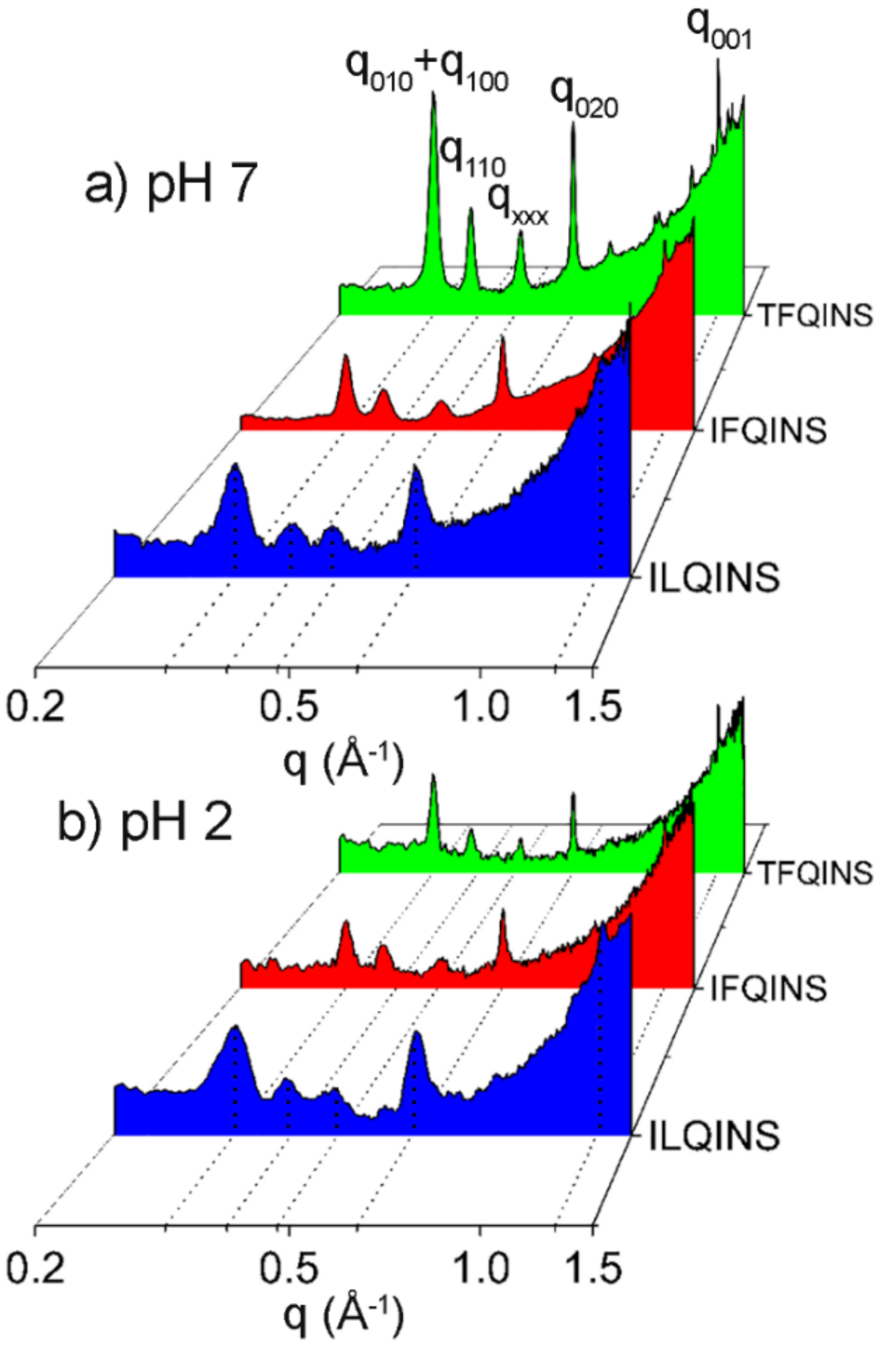

Supplementary Figure 8: Wide Angle X-ray Scattering Spectra of the three peptides at 1.5 mM and at a) pH 7 and b) pH 2 after 24 h assembly.

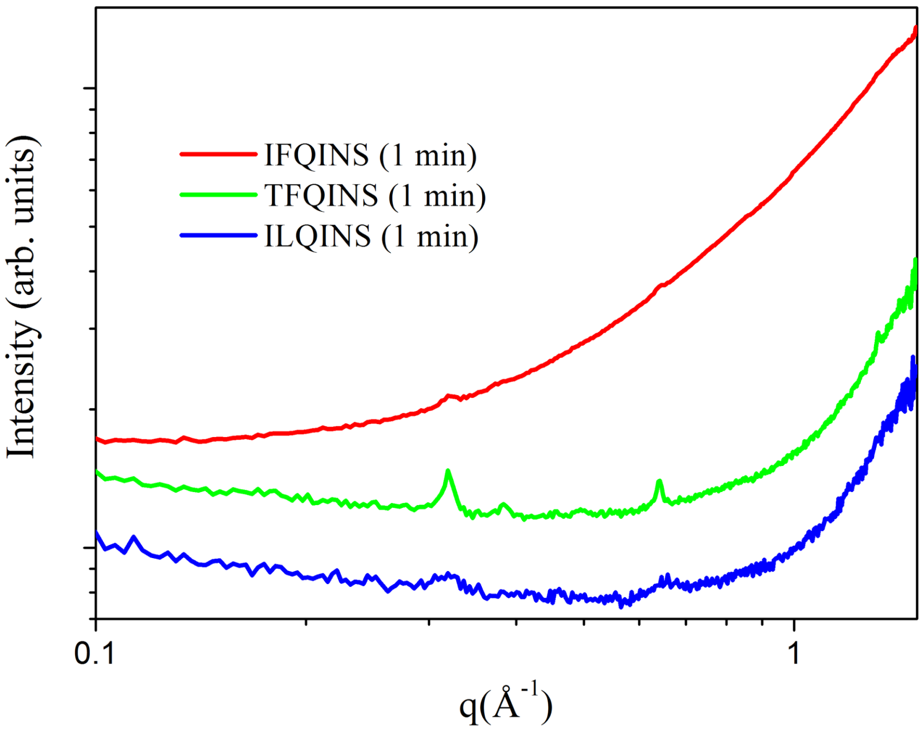

Supplementary Figure 9: WAXS spectra after one minute of the 3 hexapeptides, all 3 have very fast assembly kinetics with some evidence of Bragg peaks after 60 s, TFQINS shows more intense peaks.

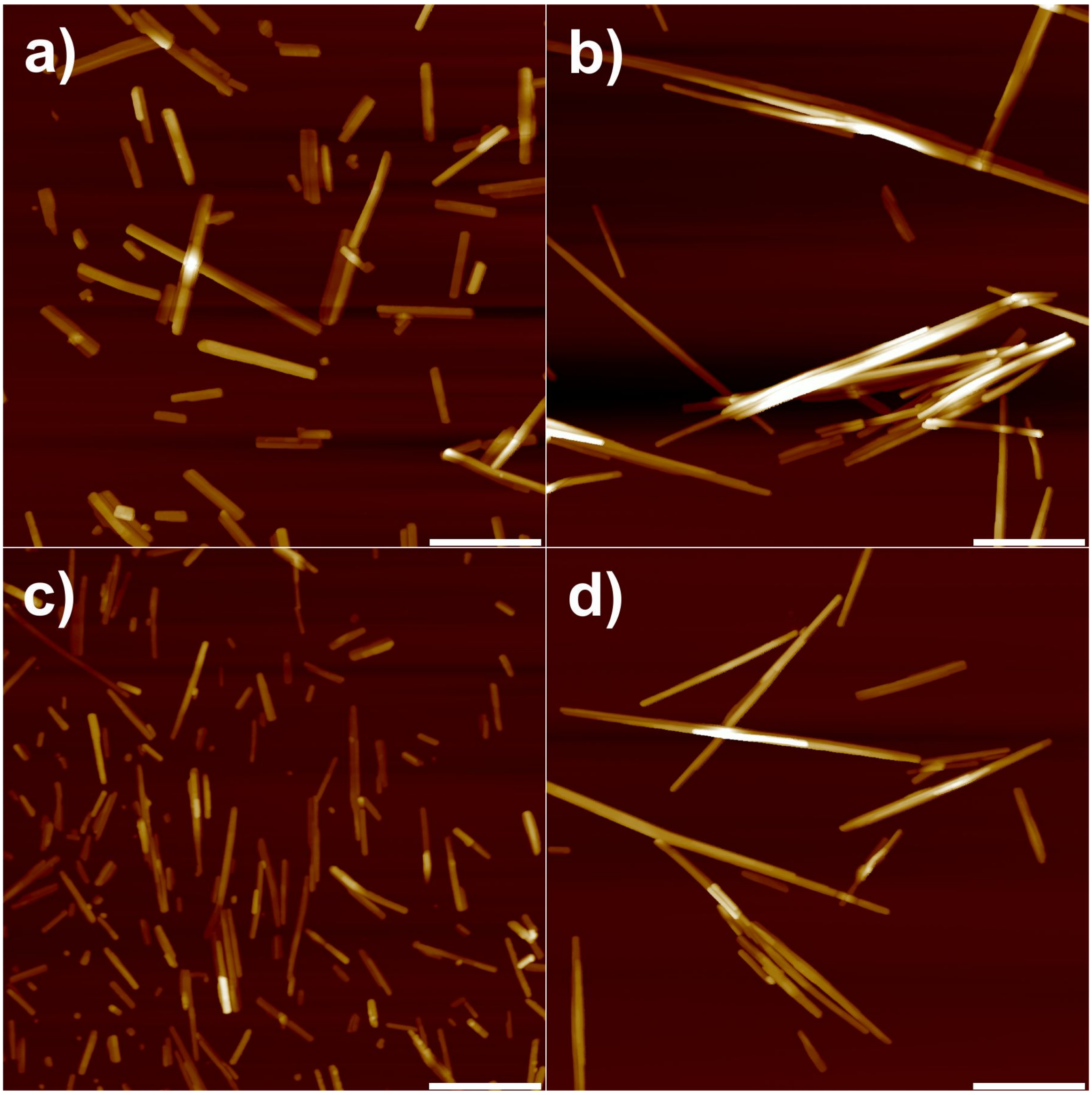

Supplementary Figure 10: AFM images of TFQINS assembly at (a) 10 min at pH 2, (b) 1 h at pH 2, (c) 10 min at pH 7, (d) 1 h at pH 7 deposited on mica. All scale bars = 600 nm, all z-scales = 75 nm

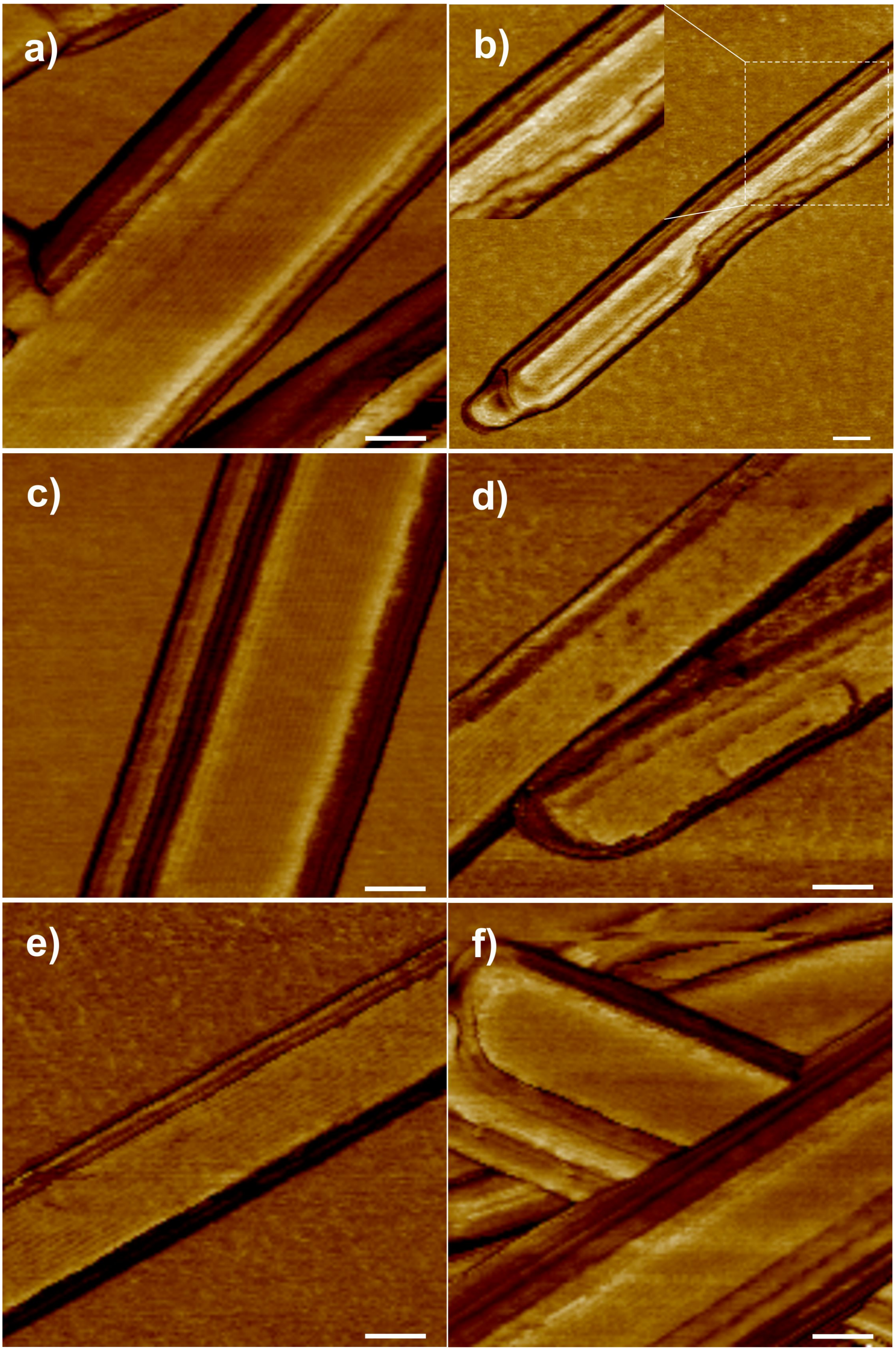

Supplementary Figure 11: (a-f) Additional AFM phase images of TFQINS crystals deposited on mica (all scale bars = 20 nm).

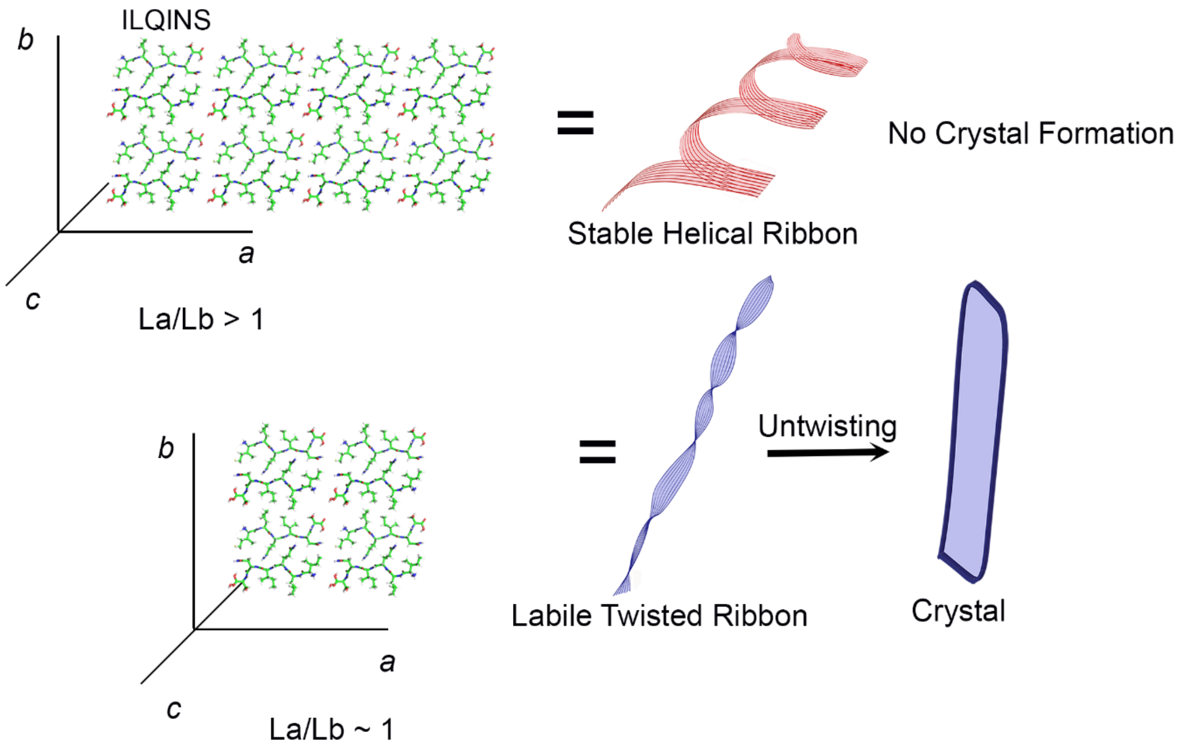

Supplementary Figure 12: Cartoon showing the two assembly pathways available and how they relate to the cross-sectional aspect ratio of amyloid oligomers.

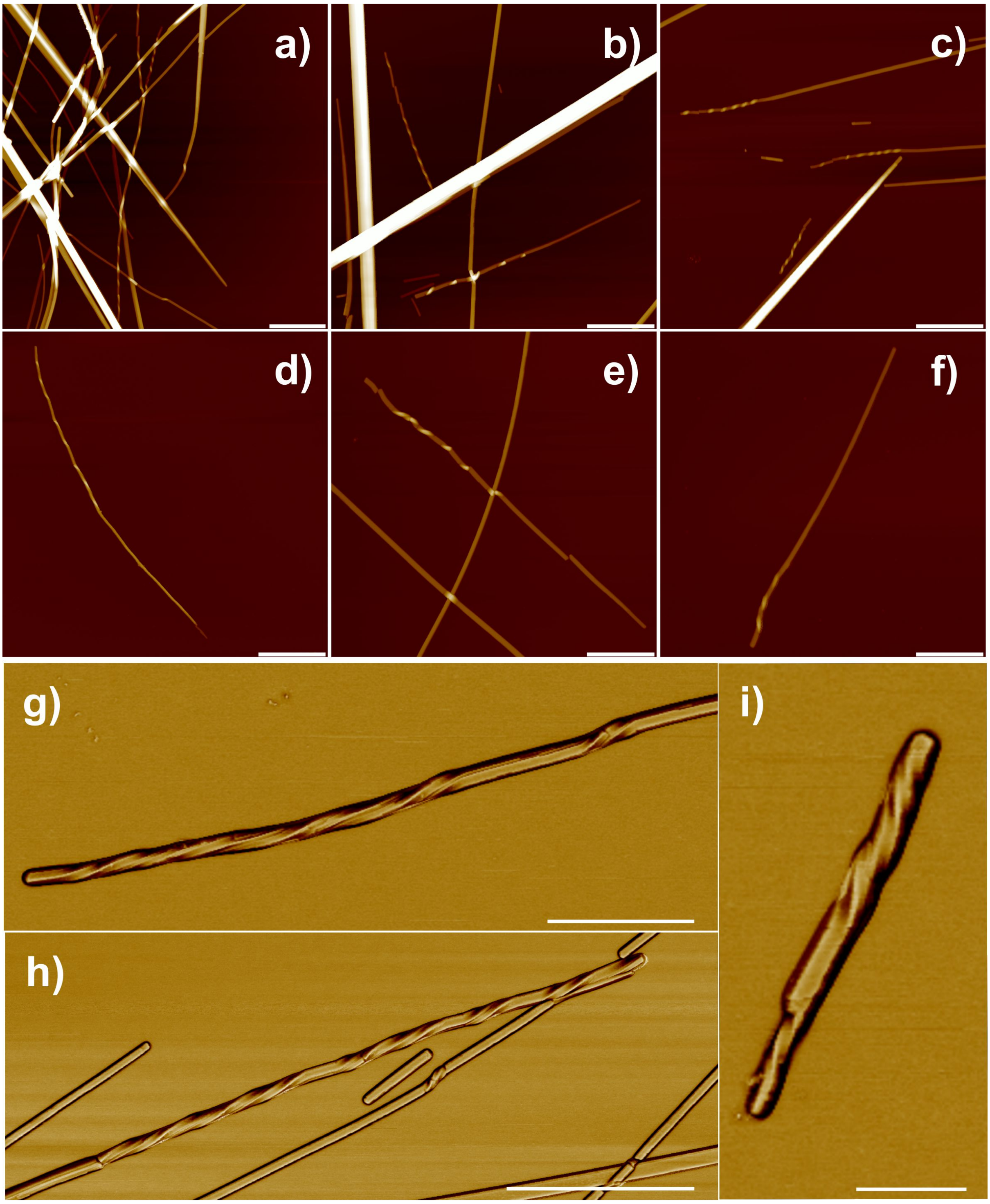

Supplementary Figure 13: (a-f) AFM images and (g-i) AFM phase images of TFQINS assembly at pH7 at higher concentration (5 mM) deposited on mica. (a-d) Scale bars = 1µm, (e) scale bar = 600 nm, (f) scale bar = 500 nm, (a-f) z-scale = 75 nm, (g) scale bar = 400 nm, (h) scale bar = 800 nm, (i) scale bar = 150 nm.

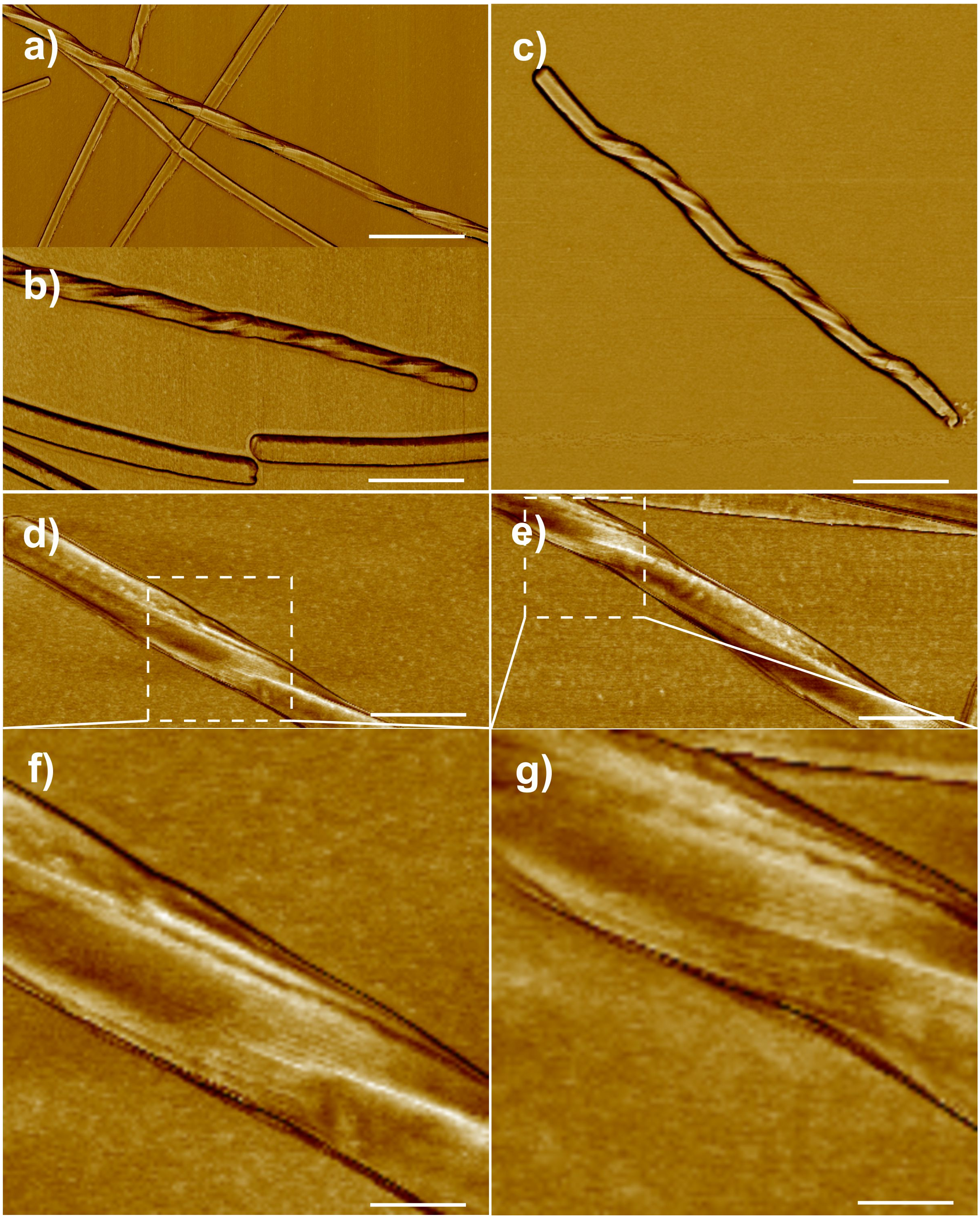

Supplementary Figure 14: (a-g) AFM phase images of TFQINS assembly at pH7 at higher concentration 5 mM deposited on mica. (a) scale bar = 300 nm, (b) scale bar = 150 nm, (c) scale bar = 200 nm, (d,e) scale bar = 75 nm, (f) scale bar = 25 nm, (g) scale bar = 20 nm.

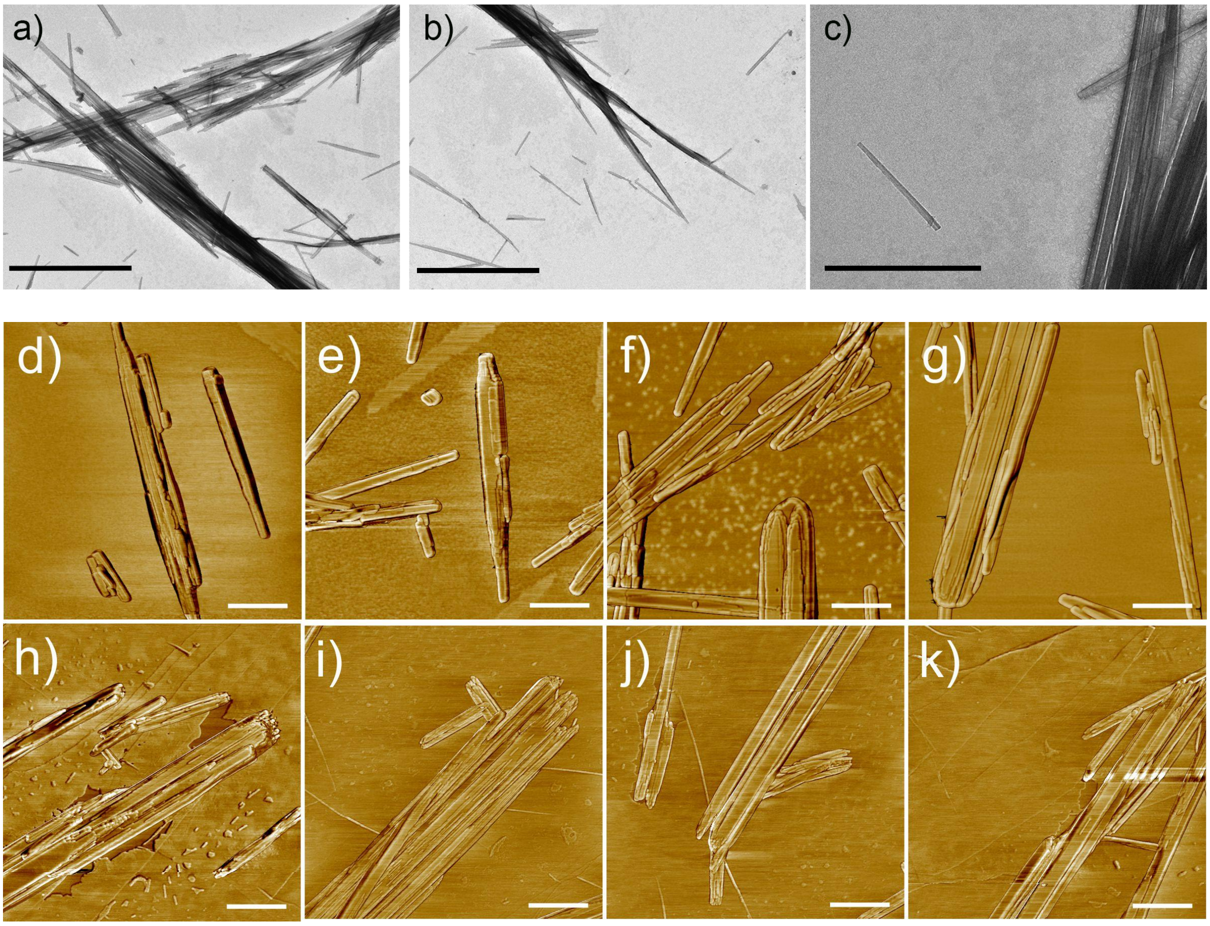

Supplementary Figure 15: Lateral Aggregation of TFQINS crystals at pH7 TEM (a-c) and AFM phase images on mica (d-g) and on HOPG (h-k). (a,b) scale bars = 5 µm, (c) scale bar = 2µm, (d-k) (scale bar = 400 nm).

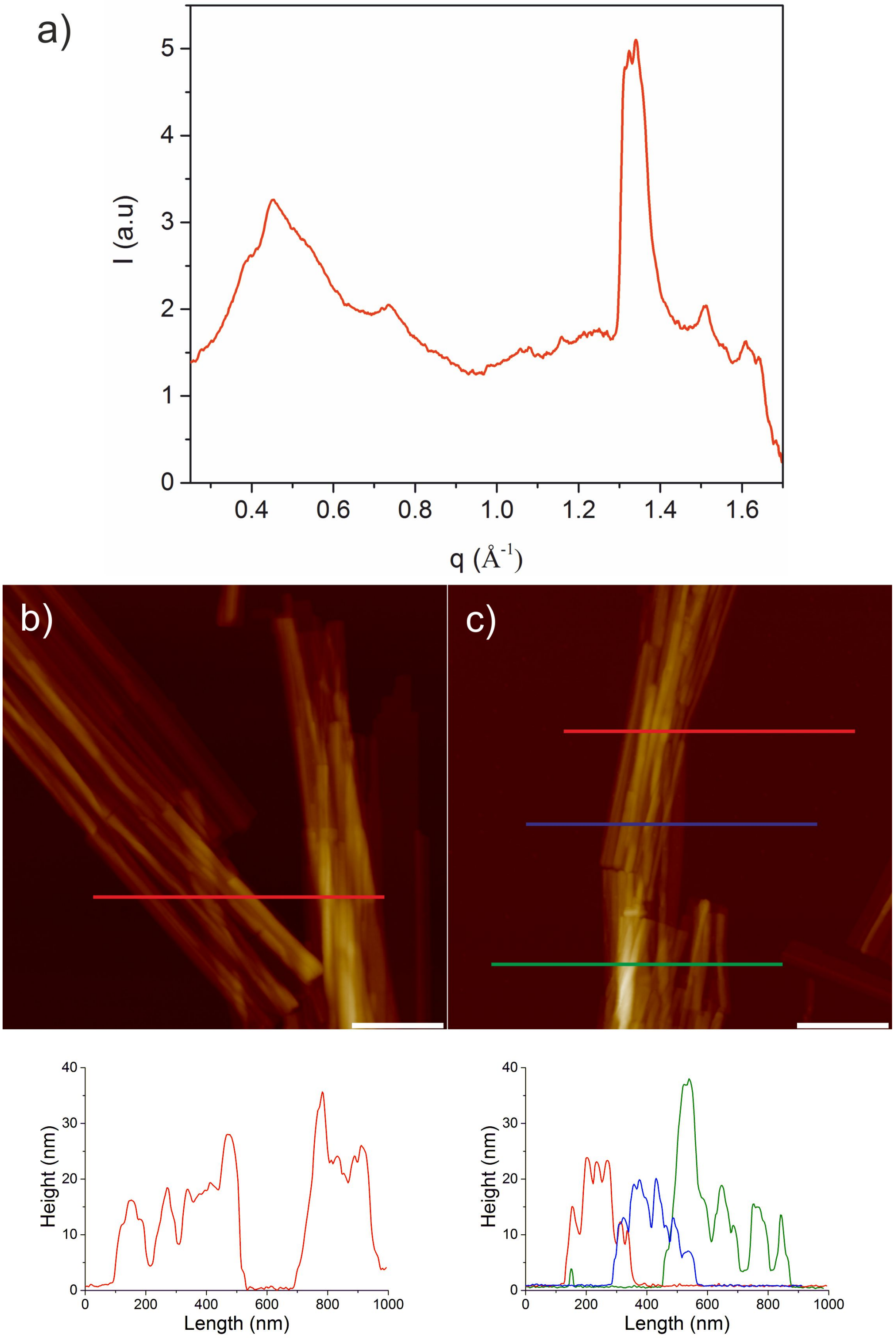

Supplementary figure 16: Figure S16: Evidence of crystal formation from longer (26mer) R3 peptide sequence, a) WAXS of solutions of assembled peptide showing multiple Bragg reflections in addition to the expected intersheet and interstrand reflections, and b) and c) AFM images of crystalline species with the corresponding height profiles. All scale bars = 300 nm, z-scale = 50 nm.

Supplementary Tables:

Supplementary Table 1: Mean values of contour length and width extracted from the statistical analysis of crystals (c) formed at 1.5 mM the values highlighted in bold represent species trapped in a fibrillar (f) state.

	Contour Length (nm)		Width (nm)	
	pH2	pH7	pH2	pH7
ILQINS	804 (c)	**1722 (f)**	53.2(c)	**27.0 (f)**
IFQINS	1280 (c)	1394 (c)	87.2 (c)	89.0 (c)
TFQINS	1606 (c)	1797(c)	186.9 (c)	164.5 (c)

Supplementary Table 2: Signal to Noise (S:N) ratios of the largest WAXS reflection (0.32 $Å^{-1}$) from the three peptides at pH 7 and pH 2

	(S:N)	
	pH2	pH7
ILQINS	1.70	1.55
IFQINS	1.36	1.49
TFQINS	1.92	4.58

Supplementary Table 3: Standard solvation free energy gain to construct a buried interface in the direction of peptide chains (ΔG_a^o) or sidechains (ΔG_b^o). Construction of buried interfaces in c (fibril axis direction) was dramatically favorable for all systems. Units are kcal/mol/(peptides buried by the interface).

	ΔG_a^o	ΔG_b^o	ΔG_c^o
ILQINS pH 7	-1.35(1)	-2.05(1)	-16.28(1)
IFQINS pH 7	-1.10(1)	-2.01(1)	-16.60(2)
TFQINS pH 7	-0.67(1)	-1.92(1)	-14.82(2)
ILQINS pH 2	-0.60(1)	-1.44(1)	-17.23(2)
IFQINS pH 2	-0.39(1)	-1.46(1)	-17.29(2)
TFQINS pH 2	-0.45(1)	-1.51(1)	-16.00(2)

Supplementary References:

1 Usov, I. & Mezzenga, R. FiberApp: An open-source software for tracking and analyzing polymers, filaments, biomacromolecules, and fibrous objects. *Macromolecules* **48**, 1269-1280, (2015).

2 Svergun, D., Barberato, C. & Koch, M. H. J. CRYSOL - A program to evaluate x-ray solution scattering of biological macromolecules from atomic coordinates. *J. Appl. Crystallogr.* **28**, 768-773, (1995).

3 Ortega, A. & de la Torre, J. G. Hydrodynamic properties of rodlike and disklike particles in dilute solution. *J. Chem. Phys.* **119**, 9914-9919, (2003).

4 Buell, A. K. *et al.* Detailed Analysis of the Energy Barriers for Amyloid Fibril Growth. *Angew. Chem., Int. Ed.* **51**, 5247-5251, (2012).

5 Case, D. A. *et al. AMBER 2016*. (University of California, San Francisco, 2016).

6 Mongan, J., Simmerling, C., McCammon, J. A., Case, D. A. & Onufriev, A. Generalized born model with a simple, robust molecular volume correction. *J. Chem. Theory Comput.* **3**, 156-169, (2007).

7 Nguyen, H., Roe, D. R. & Simmerling, C. Improved generalized born solvent model parameters for protein simulations. *J. Chem. Theory Comput.* **9**, 2020-2034, (2013).

8 Varrette, S., Bouvry, P., Cartiaux, H. & Georgatos, F. Management of an Academic HPC Cluster: The UL Experience. *Proc. of the 2014 Intl. Conf. on High Performance Computing & Simulation* **IEEE**, 959-967, (2014).

9 Case, D. *et al.* AMBER 2015. *University of California, San Francisco*, (2015).

10 Lara, C. *et al.* ILQINS Hexapeptide, identified in lysozyme left-handed helical ribbons and nanotubes, forms right-handed helical ribbons and crystals. *J. Am. Chem. Soc.* **136**, 4732-4739, (2014).

11 Berendsen, H. J. C., Postma, J. P. M., Vangunsteren, W. F., Dinola, A. & Haak, J. R. Molecular-dynamics with coupling to an external bath. *J. Chem. Phys.* **81**, 3684-3690, (1984).

12 Jorgensen, W. L., Chandrasekhar, J., Madura, J. D., Impey, R. W. & Klein, M. L. Comparison of simple potential functions for simulating liquid water. *J. Chem. Phys.* **79**, 926-935, (1983).

13 DeLano, W. L. The PyMOL Molecular Graphics System. *DeLano Scientific, Palo Alto, CA, USA*, (2002).
14 Roe, D. R. & Cheatham, T. E. PTRAJ and CPPTRAJ: Software for processing and analysis of molecular dynamics trajectory data. *J. Chem. Theory Comput.* **9**, 3084-3095, (2013).
15 Hawkins, G. D., Cramer, C. J. & Truhlar, D. G. Parametrized models of aqueous free energies of solvation based on pairwise descreening of solute atomic charges from a dielectric medium. *J. Chem. Phys.* **100**, 19824-19839, (1996).
16 Gillespie, D. T. Stochastic simulation of chemical kinetics. *Annu. Rev. Phys. Chem.* **58**, 35-55 (2007).
