# Supplementary figures and images for: Competition between crystal and fibril formation in molecular mutations of amyloidogenic peptides
Source: Nat Commun. 2017 Nov 7;8:1338. doi: 10.1038/s41467-017-01424-4 (PMC5673901; doi:10.1038/s41467-017-01424-4)

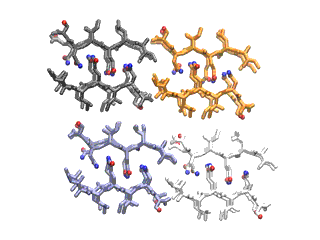

Supplement: Supplementary file 4 — Supplementary Movie 1 [file 41467_2017_1424_MOESM4_ESM.gif]

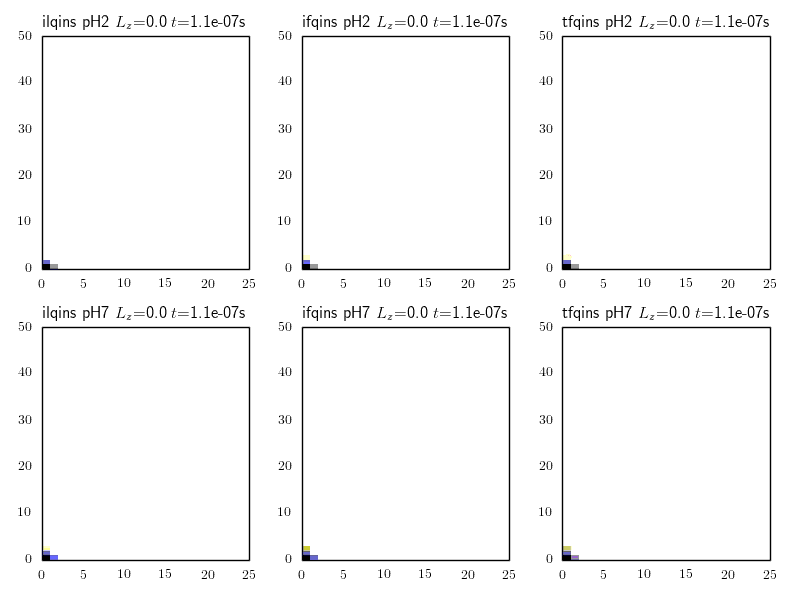

Supplement: Supplementary file 5 — Supplementary Movie 2 [file 41467_2017_1424_MOESM5_ESM.gif]

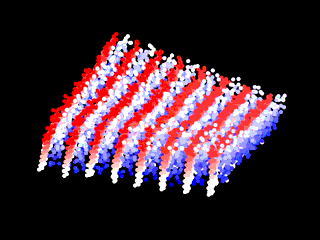

Supplement: Supplementary file 6 — Supplementary Movie 3 [file 41467_2017_1424_MOESM6_ESM.gif]
